# Eco-friendly colorimetric detection of lead and mercury using l-cysteine-functionalized gold nanoparticles: a step towards greening the exposome†

**DOI:** 10.1039/d5ra01445j

**Published:** 2025-04-14

**Authors:** Sharmila Sajankila Nadumane, Rajib Biswas, Nirmal Mazumder

**Affiliations:** a Department of Biophysics, Manipal School of Life Sciences, Manipal Academy of Higher Education Manipal Karnataka India-576104 nirmal.mazumder@manipal.edu; b Department of Physics, Tezpur University Tezpur Assam India-784028 rajib@tezu.ernet.in

## Abstract

Heavy metal toxicity, particularly from lead (Pb) and mercury (Hg), poses a significant threat to the biological system and the exposome, even at trace levels. In alignment with the ‘Greening the Exposome’ initiative, this study presents an eco-friendly and rapid colorimetric technique for detecting Pb and Hg in water. This method utilizes biogenic l-cysteine functionalized gold nanoparticles (AuNPs) of varying sizes as a colorimetric sensor, offering selective detection of these heavy metals. The utilization of l-cysteine, a naturally occurring amino acid, enhances the sustainability of the assay. The l-cysteine functionalized AuNPs demonstrate selectivity towards Pb and Hg through visually observable color changes, directly correlated with shifts in the surface plasmon resonance (SPR). The characteristic SPR peak of AuNPs, initially observed around 525 nm, undergoes a red shift upon aggregation in the presence of Pb and Hg, resulting in peaks at approximately 725 nm for Pb and 700 nm for Hg. The calibration curve shows a linearity range of 100–500 ppb for determination of Pb and Hg with a limit of detection of 290 ppb and 140.35 ppb. This simple, cost-effective, and environmentally conscious approach offers a promising tool for monitoring heavy metal contamination in aqueous environments, contributing to a better understanding and management of the chemical exposome.

## Abstract 2

Representation of sources of Pb and Hg contamination and colorimetric detection using AuNPs modified with l-cysteine.

## Graphical abstract

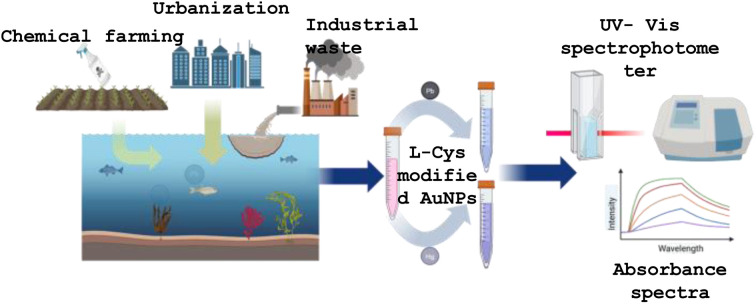

## 1. Introduction

Heavy metal contamination in aquatic environments poses a significant threat to human health and ecosystems due to its persistence, bioaccumulation, and toxicity. Industrial discharge, mining activities, and agricultural runoff contribute to elevated levels of hazardous metals such as lead (Pb^2+^), cadmium (Cd^2+^), mercury (Hg^2+^), and arsenic (As^3+^) in water bodies, necessitating sensitive and reliable monitoring strategies.^1^ Traditional detection techniques, including atomic absorption spectroscopy (AAS) and inductively coupled plasma mass spectrometry (ICP-MS), offer high sensitivity but are often hindered by high costs, complex sample preparation, and environmental concerns related to reagent consumption and waste generation. Increased anthropogenic activity has led to higher levels of these hazardous metals in the environment, where they enter the food chain and cause adverse effects on human health.^2^ Among these heavy metals, Hg and Pb are considered as the most toxic, with their permissible limit of 6 and 10 μg L^−1^ respectively in water. Exposure to these heavy metals is unavoidable for humans, animals and plants. Major sources of low dose human exposure include dental amalgams^3^ paints, automobile exhausts *etc.* Pb can adversely affect the human body even at very low concentrations, causing saturnism, that mainly affects the nervous and gastrointestinal systems.^4^ Prolonged Hg exposure leads to Minamata disease, which damages the cerebellar cortex and peripheral sensory nerves. The most frequently used techniques for detecting heavy metals include analytical methods such as ICPMS,^5^ UV-vis spectroscopy, Surface-Enhanced Raman Scattering (SERS),^6^ AAS,^7^ X-ray fluorescence (XRF).^8^Table 1 represents the various types of techniques used in the detection of heavy metals with their Limit of Detection (LODs). Various colorimetric approaches include catalysis by nanozymes,^9^ surface plasmon resonance,^10^ ligand receptor binding and fluorescent colorimetric detection.^11^ These can be applied according to the analyte and the detection requirements.^8^ Nanomaterials like metal nanoparticles,^11,12^ metal oxides,^13^ sulphides and graphene-based nanomaterials cause a visible color change when detecting the heavy metals.^14^

**Table 1:** List of conventional techniques used in heavy metal detection

Methodology	Details	Limit of detection (LOD)	Reference
UV-vis spectroscopy	UV-vis spectroscopy measures absorbance changes	1.0 × 10^−7^ M (11.24 ppb)	22
Surface-enhanced Raman scattering (SERS)	SERS enhances Raman signals using nanostructures for sensitive detection of heavy metals.	1.6 μg L^−1^ (1.6 ppb)	23
Inductively coupled plasma mass spectrometry (ICP-MS)	A highly sensitive technique that ionizes samples using inductively coupled plasma and detects metal ions *via* mass spectrometry	0.001 ppb	24
Atomic absorption spectrometry (AAS)	Measures the absorption of light by free metal ions in the gaseous state; commonly used for detecting metals like mercury and lead	0.03 μg L^−1^ (0.03 ppb)	25
X-ray fluorescence (XRF)	Utilizes X-ray emission to determine elemental composition; offers rapid and non-destructive analysis	0.2 mg kg^−1^ (200 ppb)	26

In response to the growing need for sustainable and green analytical approaches, electrochemical sensing has emerged as a powerful alternative for detecting heavy metal ions in aqueous solutions. Electrochemical sensors offer advantages such as low energy consumption, minimal reagent use, portability, and rapid analysis, making them ideal for real-time environmental monitoring. Furthermore, the integration of bio-based materials, nanostructured electrodes, and green synthesis approaches in sensor fabrication enhances sensitivity while ensuring environmental sustainability.

Recent advancements in nanomaterial-based sensors focus on optical sensors, including fluorescence, SERS and Surface Plasmon Resonance (SPR) sensors. Plasmonic structures such as silver nanoparticle (AgNP) and gold nanoparticle (AuNP) exhibit SPR properties suitable for colorimetric detection.^15^ Changes in their SPR properties cause a visual colour change. AuNPs are particularly well-suited for colorimetric detection due to their stability, strong SPR, and distinct color change from red to blue compared to AgNPs.^16^ They are also easy to synthesize and have high absorption coefficient (108 cm M^−1^ for AuNPs), they also have higher surface to volume ratio for efficient target binding capacity.^17^ The surface of AuNP can be easily modified by ligands, such as thiol and amine groups through Au–S or Au–N bonds.^18^ This expands their ability to detect various compounds and enhance their sensitivity. Hence, AuNPs are highly suitable for functionalization and for use of colorimetric detection.^19^ Zhou *et al.* developed a centrifugal microfluidic system for simultaneous detection of Pb^2+^,Hg^2+^ and As^3+^ using aptamer-based colorimetric method. In the presence of specific heavy metals, the aptamer triggered AuNP aggregation, leading to a detectable color change. The LOD of Pb^2+^, Hg^2+^ and As^3+^ were 6.16 ppb, 4.97 ppb and 5.24 ppb respectively.^20^ Yuan developed a method based on microfluidic electrophoresis with indirect chemiluminescence for simultaneous detection of Cd^2+^, Pb^2+^ and Hg^2+^. In this method, Co^2+^ was used as the probe ion. The mixture of luminol and hydrogen peroxide and Co^2+^ were passed through the mixer of microfluidic channel. The method involved the displacement of probe ion by Cd^2+^, Pb^2+^ and Hg^2+^. The LOD was found to be 5.83 × 10^−8^ M, 5.83 × 10^−8^ M and 2.09 × 10^−8^ M respectively.^21^ In the present study, we have surface modified AuNPs using l-cysteine (l-Cys) for selective detection of Pb and Hg. The method is simple, involving only two steps synthesis of AuNPs and surface functionalization using l-Cys. The use of l-Cys, a biodegradable amino acid as functionalizing agent minimizes the need for the hazardous chemical reagent such as 3-mercaptopropionic acid (3-MPA) or use of chelating reagents such as ethylenediaminetetraacetic acid (EDTA). The resulting l-Cys modified AuNPs acts as a colorimetric sensor that can be used to quantify heavy metal.

Accordingly, this study explores the development of an eco-friendly sensing scheme for the detection of heavy metal ions in aqueous solutions. By leveraging sustainable biomaterials mediated plasmonic nanoparticles and optimized sensor architectures, we aim to contribute to the global effort of “greening the exposome” through environmentally benign and accessible analytical methodologies.

## 2. Materials and method

### 2.1. Materials and chemicals

Gold chloride (HAuCl_4_) (Sigma Aldrich, United States), trisodium citrate (Sigma Aldrich, United States), l-cysteine (Loba Chemicals, India), arsenic, cadmium, lead, mercury (Loba chemicals, India), magnetic stirrer (IKA India Private Limited), hydrochloric acid (Loba Chemicals, India), nitric acid (Sigma Aldrich, United States).

### 2.2. Instrumentation

We have taken the absorption reading of nanoparticles using UV-vis spectrophotometer (Tecan Spark M20, Austria). The size of AuNPs were measured using zeta sizer (Malver Zeta sizer Nano ZS Analyzer, United Kingdom). The pH of the solution was measured using pH meter (Eutech Bench Meter-ECPHTUTOR-S, Singapore).

### 2.3. Synthesis of l-Cys modified AuNPs

The widely used Turkevich method was employed for the synthesis of AuNPs in the range of 15–30 nm that provides the advantage of controlling the size and reproducibility.^27^ The AuNPs were synthesized using HAuCl_4_ where trisodium citrate was used as reducing agent.^28^ The glassware used in the synthesis was cleaned with aqua regia, a 7 : 3 mixture of HCl and HNO_3_. Further, they were rinsed with distilled water and thoroughly dried in a hot air oven. 20 mL of 195 μM HAuCl_4_ was taken in 200 mL conical flask and heated at 100 °C on a magnetic stirrer till boiling. To avoid the contamination, the conical flask was closed using a Petri dish. When the HAuCl_4_ solution started boiling, 270 μl trisodium citrate (0.1 M) was added dropwise with stirring. The solution was stirred at the same temperature until the color of the solution changed from yellow to cherry red. The conical flask was then set to cool to room temperature.^29^ The size of the synthesized nanoparticles was measured using zeta sizer. The AuNPs were centrifuged at 12 000 Revolutions Per Minute (RPM) for 15 min, and the supernatant was discarded to remove extra citrate. The AuNPs are then redispersed with 20 mL of MilliQ. To 20 mL of AuNP solution 400 μL of l-Cys (0.02 mM) was added dropwise and the mixture was stirred for 2 h to facilitate surface functionalization. The functionalized AuNPs were centrifuged to remove the extra l-Cys and MilliQ water was added to bring the solution to the desired volume. The solution was stored until further analysis.

## 3. Results and discussion

### 3.1. Characterization of the synthesized AuNP

**Fig. 1:**
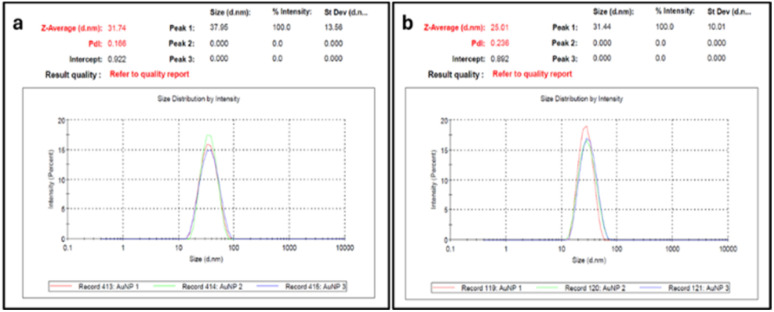
Particle size measurement of synthesized AuNPs of average size (a) 31 nm and (b) 25 nm.

The absorbance and size of the synthesized AuNPs were measured using a UV-vis spectrometer and zeta sizer respectively. The average size of the nanoparticles was found to be 31 nm and 25 nm (Fig. 1a and b).

**Fig. 2:**
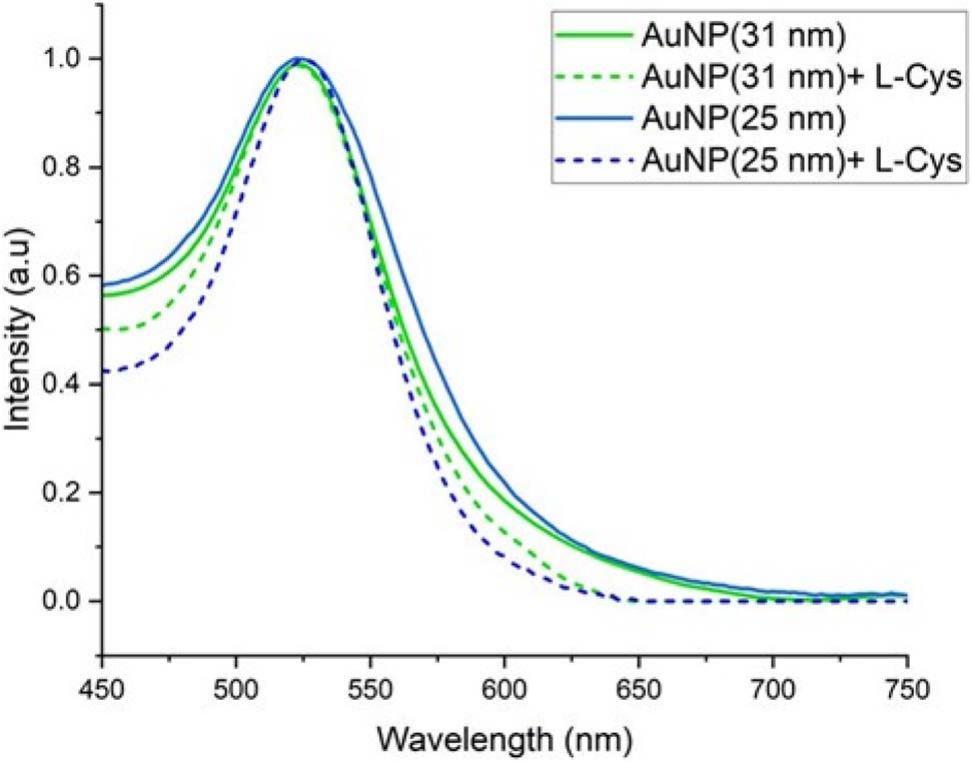
Absorbance spectra of synthesized AuNPs with and without l-Cys.

The absorbance maxima of the synthesized AuNPs and l-Cys functionalized nanoparticles were around 525 nm, which is the characteristic wavelength of AuNPs. However, the functionalized nanoparticles showed narrower peaks compared to the unfunctionalized AuNPs (Fig. 2). The thiol group of l-Cys interacts with gold and stabilizes the nanoparticles by preventing the aggregation resulting in uniformly sized nanoparticles with sharp and narrow absorbance peaks. The color variation of the nanoparticles is due to the SPR properties, where nanoparticles size influences the peak wavelength shift. Larger nanoparticles cause the SPR peak to shift towards higher wavelengths (red shift), while smaller nanoparticles display the SPR peak at shorter wavelengths.^30^ Additionally, larger nanoparticles produce broader peaks with reduced intensity, whereas smaller nanoparticles result in sharper peaks.

### 3.2. Selectivity of l-Cys functionalized AuNPs towards Pb and Hg detection

**Fig. 3:**
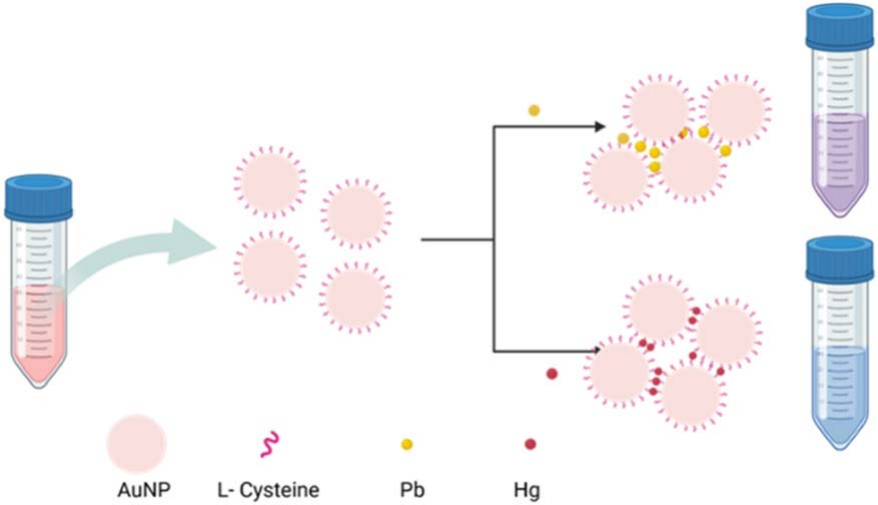
Schematic representation of the binding of l-Cys on the surface of AuNP and the interaction with Pb and Hg.

**Fig. 4:**
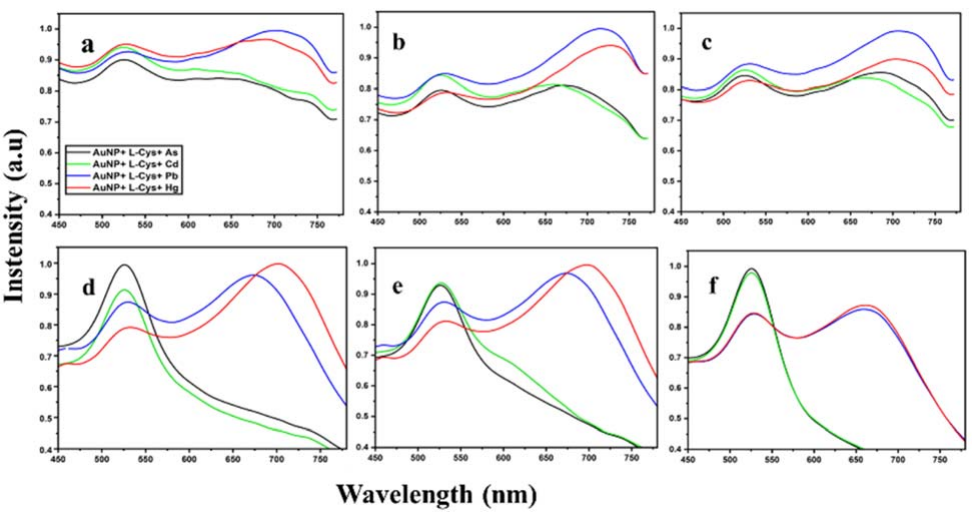
Wavelength *vs.* intensity graph of gold nanoparticles (AuNPs) functionalized with l-cysteine. The top row presents data for 31 nm AuNPs at pH levels of 6.2 (a), 7.0 (b), and 9.0 (c). The bottom row displays corresponding data for 25 nm AuNPs at pH levels of 6.2 (d), 7.0 (e), and 9.0 (f).

Biological components such as peptides and amino acid including DNAzymes, thymine and l-Cys, have shown significant selectivity in detecting Hg.^31^ The amino acid l-Cys is a neutral polar molecule that contains three different functional groups: thiols (–SH), amine (–NH_2_) and carboxyl (–COOH). The –SH group forms a stable bond with AuNPs, making it ideal for functionalization.^32^l-Cys is widely used to functionalize AuNP for detecting heavy metals, nucleobases and aptamers, citrate, glutathione, and ascorbate.^33–35^ Among the major heavy metals, Hg, Pb and Cd exhibit a higher affinity for –SH groups. Assessing the selectivity of functionalized AuNPs for specific heavy metals is essential for accurately detecting their concentrations within mixtures. In this study, we evaluated the selectivity of l-Cys functionalized AuNPs for detecting As, Cd, Pb, and Hg at a concentration of 500 ppb. AuNPs show greater stability at pH ranging from 5 to 9. However, the addition of heavy metals disrupts stability due to the interaction between nanoparticles and heavy metals resulting in aggregation (Fig. 3). In this experiment, we have analyzed the interaction between surface-functionalized AuNPs and heavy metals at three different pH (6.2, 7.0 and 9.0). The pH of the AuNP solution was measured immediately after functionalization and found to be around 6.2. Subsequently, AuNP-l-Cys solutions with pH levels of 7 and 9 were prepared by adding NaOH solutions. For the experiment, three different pH levels (6.2, 7 and 9) of AuNP-l-Cys solutions and two different nanoparticles of average size (31 nm and 25 nm) were used. Standard heavy metal solutions with a concentration of 500 ppb (As, Cd, Pb, Hg) were prepared. To 200 μL of each 500 ppb heavy metal solution, 200 μL of functionalized AuNP solution was added and left undisturbed for 15 min to allow the binding of heavy metal ions to the functionalized AuNPs. AuNPs are an effective alternative for detecting chemical species such as heavy metals in environmental samples. These nanoparticles can be conjugated with various compounds to enhance their biocompatibility, stability, and functionality. The aggregation caused by a chemical analyte induces a red shift, observed as a distinct color change from red to blue. The presence of analyte can be confirmed either by visually observing the colour change or by measuring the absorbance wavelength using UV-vis spectroscopy.^36^ Amino acids with multiple functional groups can effectively bind to both AuNPs and other chemical compounds, resulting in altered SPR properties and making them suitable for optical detection techniques. Fig. 4a–f shows the UV-vis absorption spectra of AuNPs of average size 31 nm and 25 nm functionalized with l-Cys and pH (6.2, 7 and 9). In the case of AuNPs 31 nm, at pH 6.2 visible aggregation was observed in Pb and Hg, resulting in an additional peak around 725 nm for Pb and 700 nm for Hg (Fig. 4a). The aggregation is due to the interaction between the functionalized AuNPs and the heavy metals that results in the crosslinking and the destabilization of the nanoparticles. The mechanism involves electrostatic interactions and chelation between the particles where the functionalized nanoparticles interact with the heavy metals leading to particle aggregation by diminishing the repulsive forces between the AuNPs.^37^ The change in pH of the functionalized AuNP to 7 and 9 (Fig. 4b and c), produced sharper additional peaks of Pb and Hg compared to the 6.2 pH. The increased sharpness of the peak suggests the stronger interaction between the nanoparticles and the heavy metal ions resulting in increased aggregation efficiency of the nanoparticles. The higher pH results in the reduced stability of the nanoparticles due to the change in the surface charge and ligand availability. Unlike AuNPs of 31 nm size, the 25 nm AuNPs showed visible aggregation with Pb and Hg in all the three pH levels (6.2, 7 and 9). This shows the greater affinity of these heavy metals for the smaller nanoparticles leading to aggregation regardless of the pH. AuNPs showed very sharp peaks at 675 nm for Pb and 725 nm for Hg at pH 6.2 and 7 (Fig. 4d and e). The sharpness of these peaks suggests a well-defined plasmonic resonance associated with the aggregated state, reflecting a strong coupling between the nanoparticles. However, at pH 9, both the absorbance peaks were observed around 675 nm (Fig. 4f). The shift indicates the change in the state of aggregation or structural arrangement of the nanoparticles in response to the increased pH. The interaction between the functionalized AuNPs and both the metal ions became similar under high alkaline conditions forming the peak at same wavelength. The difference in the behaviour of the AuNPs at two different sizes (31 nm and 25 nm) with the heavy metals indicates how the size of the nanoparticles influences reactivity and interaction. Smaller nanoparticles show higher surface-area to volume ratios, that allows the more effective binding of the nanoparticles.

The synthesis using the Turkevich method was reproducible across batches, with a slight variation of ±2 nm in AuNP size. The stability of functionalized AuNPs was monitored for 5 days by measuring the absorbance and zeta potential of the l-Cys functionalized AuNPs. The table (ESI Table 1†) provides the zeta potential values of AuNPs of average size 25 nm and 31 nm for 5 days. The ESI Fig. 1–4† illustrate the absorbance of the l-Cys functionalized AuNPs and their response to heavy metal detection.

For AuNPs with average size 25 nm, the surface zeta potential remained relatively stable at pH 6.2 and 7.0. However, at pH 9, the surface zeta potential was negatively increased reaching the value −5 showing the unstable in nature. The absorbance values of AuNP-l-Cys at all three pH levels remained consistent over 5 days. Additionally, after the addition of heavy metals, the absorbance showed minimal variation, detecting Pb and Hg without any interference from other heavy metals. In case of AuNPs with average size 31 nm, it was observed that the in-surface zeta potential fluctuating between positive and negative charges. Additionally, after the addition of heavy metals, the absorbance showed the interference from As and Cd.

**Fig. 5:**
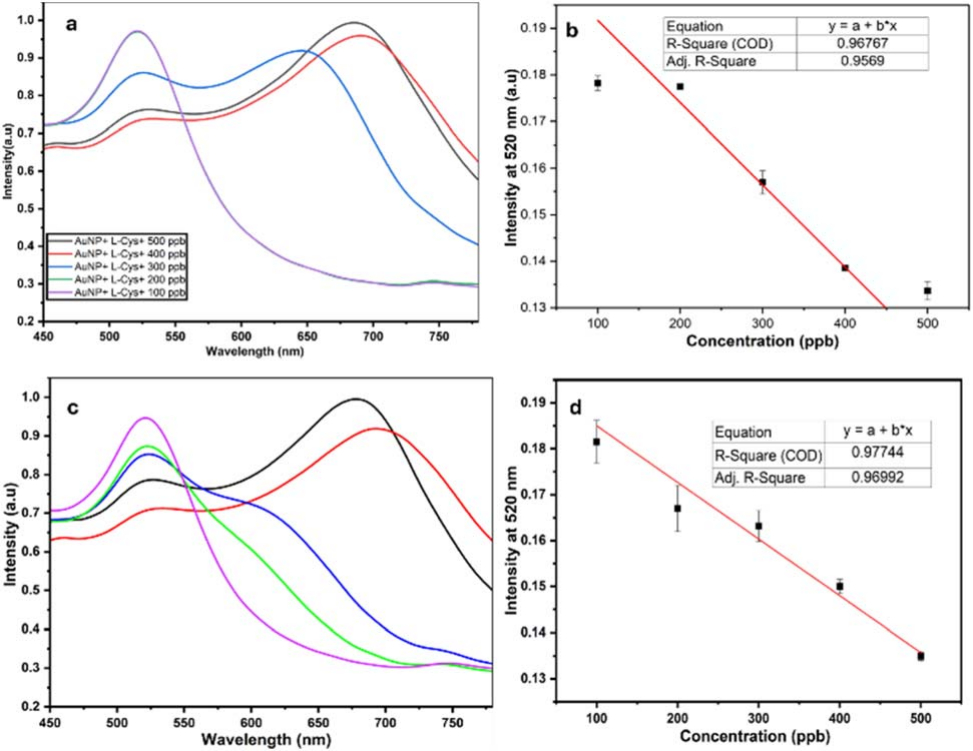
Wavelength *vs.* intensity graph of 100–500 ppb and linearity curve of Pb (a, b) and Hg (c, d).

After the analysis of absorbance of 25 nm and 31 nm AuNPs at varying pH levels, the 25 nm AuNPs exhibited distinct absorption peak at two different wavelengths for Pb and Hg. This characteristic feature helps in differentiating Pb and Hg in complex mixtures. It was observed that while using 25 nm AuNPs, interference from the other heavy metals was reduced due to the surface chemistry of the nanoparticles that shows higher affinity towards Pb and Hg compared to other heavy metals. Hg and Pb are considered as thiophilic groups that show higher affinity towards sulphur containing groups compared to other heavy metals thus forming stable bonds with l-Cys. Minimizing interference is crucial for precise detection of heavy metals particularly in environmental samples. Literature reports indicate that the stability constants for the interactions between l-Cys and Hg and Pb are relatively high as compared to other heavy metals, with log *K* values of 10.1 for Hg, 4.1 for Pb and 3.2 for Cd.^38^ Further, the Pb and Hg at concentrations ranging from 100–500 ppb were analyzed using AuNPs of average size 25 nm to determine the lowest detectable concentration. In case of Pb, the aggregation was observed in the vials containing 300 ppb, 400 ppb and 500 ppb whereas 100 ppb and 200 ppb did not show any aggregation. However, in case of Hg, 400 ppb and 500 ppb showed aggregation whereas 300 ppb and 200 ppb did not show much aggregation. A linearity was obtained in the range 100–500 ppb of Pb and Hg with a correlation coefficient of 0.96 and 0.977 respectively as shown in Fig. 5. Table 2 represents the different techniques used in the detection of heavy metals with their LOD *vis-à-vis* our work. Our work is quite comparable with these methods in terms of LOD.^39^ The table provides a comparative analysis of our method with similar studies. Our research focuses on simultaneous detection of two highly toxic heavy metals, Pb and Hg. A distinct color change from red to blue color in the vials was observed immediately upon the addition of Pb and Hg, highlighting the rapid response of our method. This enables quick, effective, naked-eye detection for identification of heavy metals.^35,39,40^

**Table 2:** Comparison of AuNP based detection methods similar to proposed study

Feature	Carbajal-Morán *et al.*, (2022)^35^	Vaid *et al.*, (2020)^34^	Sonia and Seth (2020)^40^	Our study
Synthesis of AuNPs	Laser ablation	Turkevich method	Turkevich method	Turkevich method
Functionalizing agent	l-Cysteine	Citrate, glutathione, ascorbate	l-Cysteine	l-Cysteine
Size of AuNPs	Average size-23.3 nm	Citrate-AuNPs: average size 16.3 nm, GSH-AuNPs: average size 22.4 nm, ascorbate-AuNPs: average size 8 nm	Average size-24 nm	Average size-25 nm and 31 nm
Heavy metals detected	Pb^2+^, Cd^2+^, As^3+^	Cr, Mn, Cd, Fe	Cd	Pb^2+^, Hg^2+^
Detection mechanism	UV-vis spectroscopy	UV-vis spectroscopy	UV-vis spectroscopy	UV-vis spectroscopy
SPR shift	518 nm to 700 nm	Citrate-AuNPs: in the presence of Cr, SPR shift from 521 nm to 661 nm, GSH-AuNPs: in the presence of Mn, SPR shift from 521 nm to 546 nm, ascorbate-AuNPs: in the presence of Cd, Cr, Co, Fe and Mn corresponding SPR shift were 557 nm, 551 nm, 541 nm, 555 nm, and 556 nm respectively	520–600 nm	Pb-525 nm to 725 nm, Hg-525 nm to 700 nm

### 3.3. Detection of Pb and Hg in the real water samples

The validity of the AuNP-l-Cys was tested by determining Pb and Hg in the ground water samples. 4 real ground water samples were collected from different regions in bottles and filtered using the 0.2 μm filter to remove the debris. The water samples were spiked with 500 ppb of different ratios of heavy metals at varying ratios of 1 : 1, 1 : 2, 2 : 1, 1 : 3 and 3 : 1 (heavy metals to real water samples respectively). To 400 μl of heavy metal 400 μl of AuNP-l-Cys was added. The concentration of the metal ions were determined using the standard calibration curve. The intensities and the concentrations are listed in Table 3. Based on the calculated concentrations from the calibration curve, Pb and Hg were detected at various concentrations. The variations were observed across different dilution ratios of metals indicating the importance of the composition of the water.

**Table 3:** Concentrations of Pb and Hg in real water samples

Heavy metals	Ratios (heavy metal : real water)	Sample 1		Sample 2		Sample 3		Sample 4	
		Intensity (au)	Concentration (ppb)	Intensity (au)	Concentration (ppb)	Intensity (au)	Concentration (ppb)	Intensity (au)	Concentration (ppb)
Pb	1 : 1	0.1425	401.12	0.1918	13.21	0.1878	47.94	0.1277	579.19
	1 : 2	0.1353	451.63	0.1863	61.18	0.184	82.08	0.1271	584.19
	2 : 1	0.1658	238.12	0.1866	58.83	0.1870	55.00	0.1264	590.37
	1 : 3	0.1310	481.80	0.1874	52.06	0.1845	77.66	0.1322	539.16
	3 : 1	0.1804	135.45	0.1784	131.52	0.1785	130.35	0.1271	584.49
Hg	1 : 1	0.1458	419.37	0.1899	29.98	0.1900	28.51	0.1281	575.66
	1 : 2	0.1337	525.92	0.1882	44.99	0.1888	39.11	0.1297	561.53
	2 : 1	0.1409	462.34	0.1879	47.35	0.1886	41.17	0.1325	536.81
	1 : 3	0.1353	512.08	0.1865	60.00	0.1874	51.47	0.1307	552.41
	3 : 1	0.1829	91.79	0.1884	42.64	0.1900	28.81	0.1283	573.89

## 4. Conclusion

The synthesis of AuNP is a challenging process and requires controlled conditions to achieve desired properties. In this study, Turkvish method was employed to synthesize AuNPs with an average diameter from 15–35 nm which is known for its reproducibility and in generating nanoparticles with precise size distribution, that is essential for their use in various applications including sensing of heavy metals. Among different sizes of nanoparticle synthesized, those with average size of 25 nm functionalized with 0.2 mM was considered as optimal for simultaneous detection of Pb and Hg. Furthermore, the study highlighted that the sensitivity and selectivity of l-Cys-AuNP is highly influenced by the size of the nanoparticles, the pH and the concentration of the AuNPs. The study suggests that the smaller nanoparticles provide better surface interactions with the target analytes. The application of 25 nm AuNPs in detecting Pb and Hg at varying concentrations opens opportunities for investigation into their potential as a reliable sensor for detection of heavy metals from environmental samples. Further, the technique can be applied in fabricating a simple, cost-effective device such as a paper-based colorimetric device or an optical detection system, enabling onsite detection of the heavy metals in real water samples.

## Data availability

The authors declare that data will be made available as per reasonable request to corresponding.

## Author contributions

S. S. N. conducted the experiments and wrote the manuscript. N. M. and R. B. reviewed and edited the manuscript.

## Conflicts of interest

The authors do not have any conflict of interest.

## Supplementary Material

RA-015-D5RA01445J-s001

## References

[cit1] Rahman Z., Singh V. P. (2019). Environ. Monit. Assess..

[cit2] Wu G., Kang H., Zhang X., Shao H., Chu L., Ruan C. (2010). J. Hazard. Mater..

[cit3] Berlin M. (2020). Neurotoxicology.

[cit4] Clarke E. G. C. (1973). J. Small Anim. Pract..

[cit5] Chen L., Li X., Li Z., Deng L. (2020). RSC Adv..

[cit6] Zhao Q., Zhang H., Fu H., Wei Y., Cai W. (2020). J. Hazard. Mater..

[cit7] Zhang Y., Adeloju S. B. (2012). Anal. Chim. Acta.

[cit8] Geleta G. S. (2023). Food Chem. Adv..

[cit9] Huang L., Zhu Q., Zhu J., Luo L., Pu S., Zhang W., Zhu W., Sun J., Wang J. (2019). Inorg. Chem..

[cit10] Alzahrani E. (2020). J. Anal. Methods Chem..

[cit11] Kim H. N., Ren W. X., Kim J. S., Yoon J. (2012). Chem. Soc. Rev..

[cit12] Singh H., Bamrah A., Bhardwaj S. K., Deep A., Khatri M., Brown R. J. C., Bhardwaj N., Kim K.-H. (2021). Environ. Sci.:Nano.

[cit13] Rahmati S., Doherty W., Amani Babadi A., Akmal Che Mansor M. S., Julkapli N. M., Hessel V., (Ken) Ostrikov K. (2021). Micromachines.

[cit14] Zanganeh S., Hutter G., Spitler R., Lenkov O., Mahmoudi M., Shaw A., Pajarinen J. S., Nejadnik H., Goodman S., Moseley M., Coussens L. M., Daldrup-Link H. E. (2016). Nat. Nanotechnol..

[cit15] Sabela M., Balme S., Bechelany M., Janot J., Bisetty K. (2017). Adv. Eng. Mater..

[cit16] Wilson R. (2008). Chem. Soc. Rev..

[cit17] Zhang Y., Adeloju S. B. (2012). Anal. Chim. Acta.

[cit18] Rosi N. L., Mirkin C. A. (2005). Chem. Rev..

[cit19] Du J., Jiang L., Shao Q., Liu X., Marks R. S., Ma J., Chen X. (2013). Small.

[cit20] Zhou M., Li J., Yuan S., Yang X., Lu J., Jiang B. (2024). Sens. Actuators, B.

[cit21] Yuan S. (2024). Anal. Methods.

[cit22] Xue Y., Zhao H., Wu Z., Li X., He Y., Yuan Z. (2011). Analyst.

[cit23] Ren J., Xu K., Zhang M., Zhang Q., Jing C. (2024). Sens. Actuators, B.

[cit24] Passariello B., Barbaro M., Quaresima S., Casciello A., Marabini A. (1996). Microchem. J..

[cit25] Maria G. C., D B., Mihaela I., Nicoleta P. C., Miriana G. (2013). Acta Med. Marisiensis.

[cit26] Li F., Wang J., Xu L., Wang S., Zhou M., Yin J., Lu A. (2018). Int. J. Environ. Res. Public Health.

[cit27] Yahaya M. L., Zakaria N. D., Noordin R., Abdul Razak K. (2022). Mater. Today: Proc..

[cit28] Sahu B., Kurrey R., Deb M. K., Shrivas K., Karbhal I., Khalkho B. R. (2021). RSC Adv..

[cit29] Chai F., Wang C., Wang T., Ma Z., Su Z. (2010). Nanotechnology.

[cit30] Pham L. D., Le C. H., Nguyen O. T. T., Dang C. T., Ly T. N., Nguyen N. T. T., Nguyen Van D., Thach L. T. D., Nguyen H. S. (2021). Commun. Phys..

[cit31] Liu J., Lu Y. (2007). Angew. Chem., Int. Ed..

[cit32] Rujiralai T., Leelaharat N., Cheewasedtham W. (2024). RSC Adv..

[cit33] Rossi A., Cuccioloni M., Magnaghi L. R., Biesuz R., Zannotti M., Petetta L., Angeletti M., Giovannetti R. (2022). Chemosensors.

[cit34] Vaid K., Dhiman J., Sarawagi N., Kumar V. (2020). Langmuir.

[cit35] Carbajal-Morán H., Rivera-Esteban J. M., Aldama-Reyna C. W., Mejía-Uriarte E. V. (2022). J. Ecol. Eng..

[cit36] Kusuma S. A. F., Harmonis J. A., Pratiwi R., Hasanah A. N. (2023). Sensors.

[cit37] Thuy Nguyen T. T., Han O. A., Lim E. B., Haam S., Park J.-S., Lee S.-W. (2021). RSC Adv..

[cit38] Chai F., Wang C., Wang T., Ma Z., Su Z. (2010). Nanotechnology.

[cit39] Chen Z., Zhang Z., Qi J., You J., Ma J., Chen L. (2023). J. Hazard. Mater..

[cit40] Sonia N., Seth R. (2020). Mater. Today: Proc..

