# Supplementary material for: Eco-friendly colorimetric detection of lead and mercury using l-cysteine-functionalized gold nanoparticles: a step towards greening the exposome
Source: RSC Adv. 2025 Apr 14;15(15):11573–9. doi: 10.1039/d5ra01445j (PMC11995260; doi:10.1039/d5ra01445j)
Supplement: RA-015-D5RA01445J-s001 [file RA-015-D5RA01445J-s001.pdf]

Supplementary Information (SI) for RSC Advances.
This journal is © The Royal Society of Chemistry 2025

Supplementary materials

Eco-Friendly Colorimetric Detection of Lead and Mercury Using L-Cysteine-Functionalized Gold Nanoparticles: A Step Towards Greening the Exposome

Sharmila Sajankila Nadumane[a], Rajib Biswas[b]*, Nirmal Mazumder[a] *

a.Department of Biophysics, Manipal School of Life Sciences, Manipal Academy of Higher Education, Manipal, Karnataka, India-576104
b.Department of Physics, Tezpur University, Tezpur, Assam, India-784028
c.*Corresponding authors: rajib@tezu.ernet.in ; nirmal.mazumder@manipal.edu

Supplementary table 1: Surface zeta potential values

Zeta potential (mV)						
Day	25 nm			31 nm		
	6.2 pH	7 pH	9 pH	6.2 pH	7 pH	9 pH
1	-25.7	-28.0	-15.4	-14.0	-16.6	0.167
2	-20.9	-26.8	-14.6	0.248	-0.804	-13.4
3	-25.3	-26.2	-10.1	-26.1	-13.7	-26.0
4	-19.3	-26.8	-3.90	-24.6	-14.8	-24.7
5	-22.8	-20.6	-5.39	0.199	-14.8	-23.7

Supplementary table 1: Surface zeta potential value of AuNPs of average size 25 nm and 31 nm of pH 6.2, 7 and 9) for 5 days.

Supplementary Fig 1: Stability of AuNP (31 nm)-L Cys

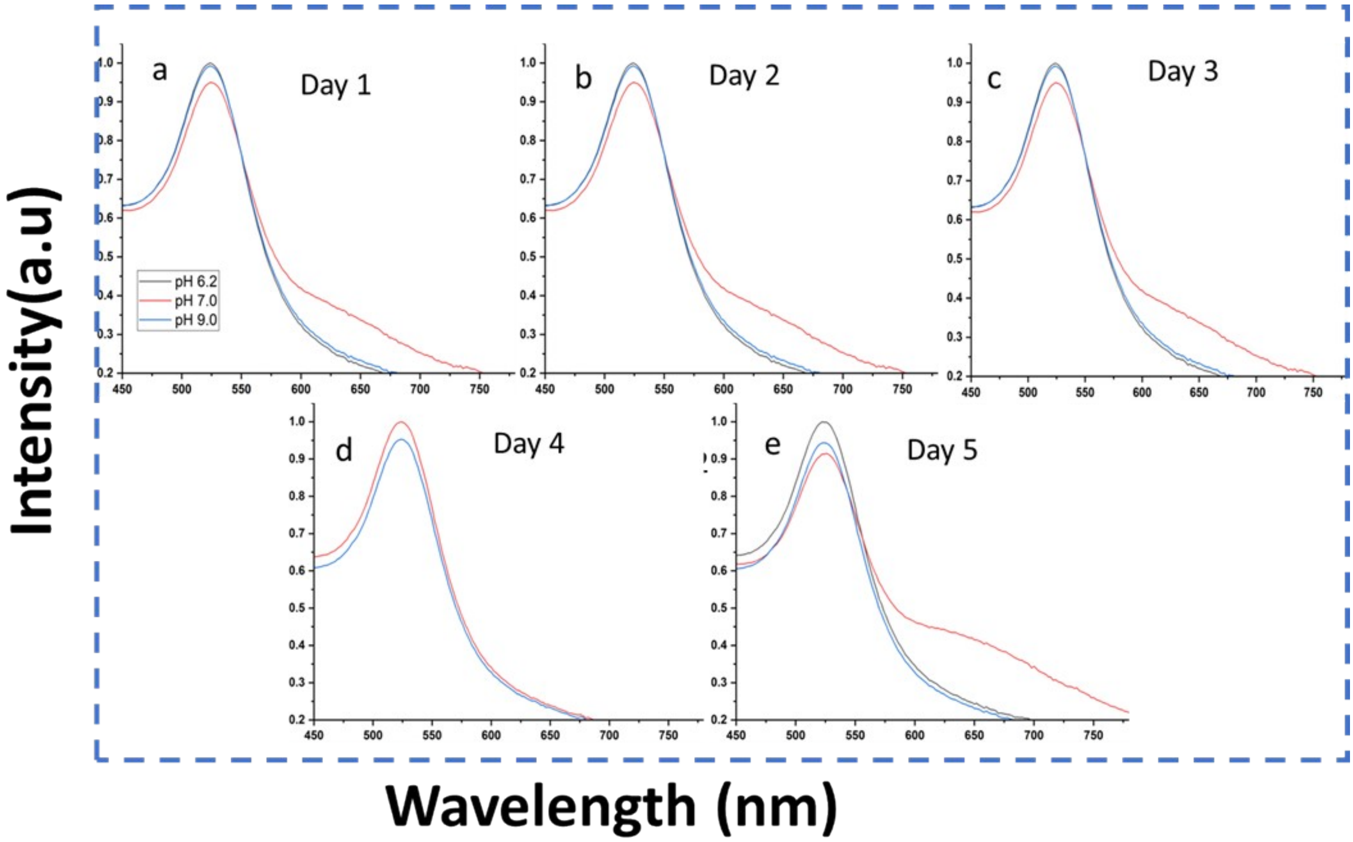

Supplementary Fig 1: Wavelength vs. intensity graph of AuNPs of average size 31 nm functionalized with L-cysteine with three different pH's 6.2, 7.0 and 9.0. for 5 days.

Supplementary Fig 2: Stability of AuNP (25 nm)-L Cys

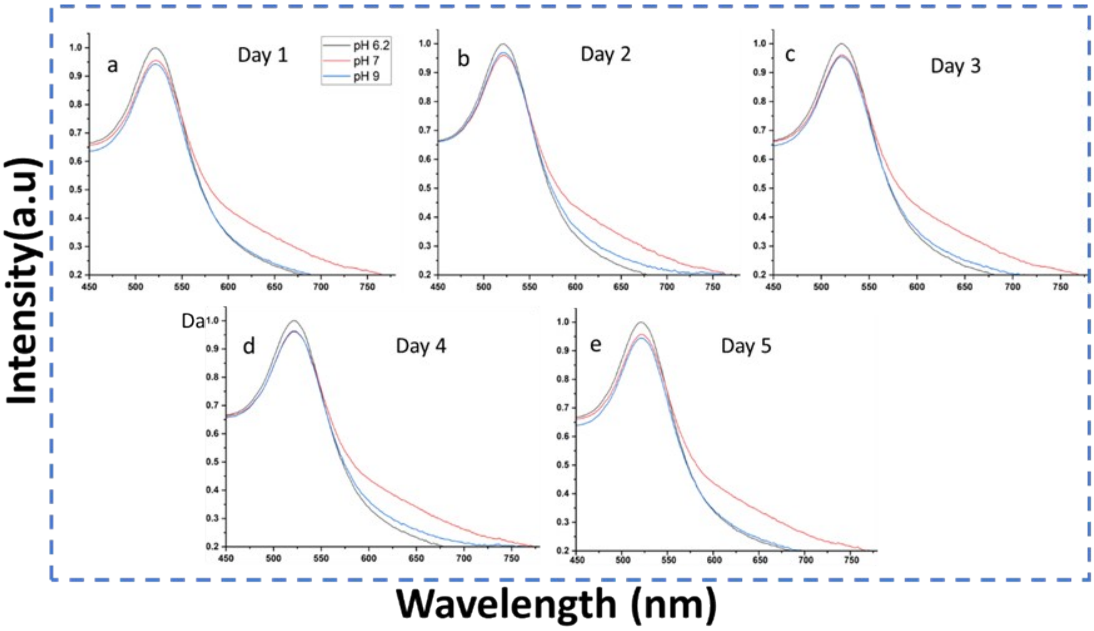

a

Supplementary Fig 2: Wavelength vs. intensity graph of AuNPs of average size 25 nm functionalized with L-cysteine with three different pH’s 6.2, 7.0 and 9.0. for 5 days.

Supplementary Fig 3: Absorbance graph of AuNP (31nm)-L Cys and heavy metals

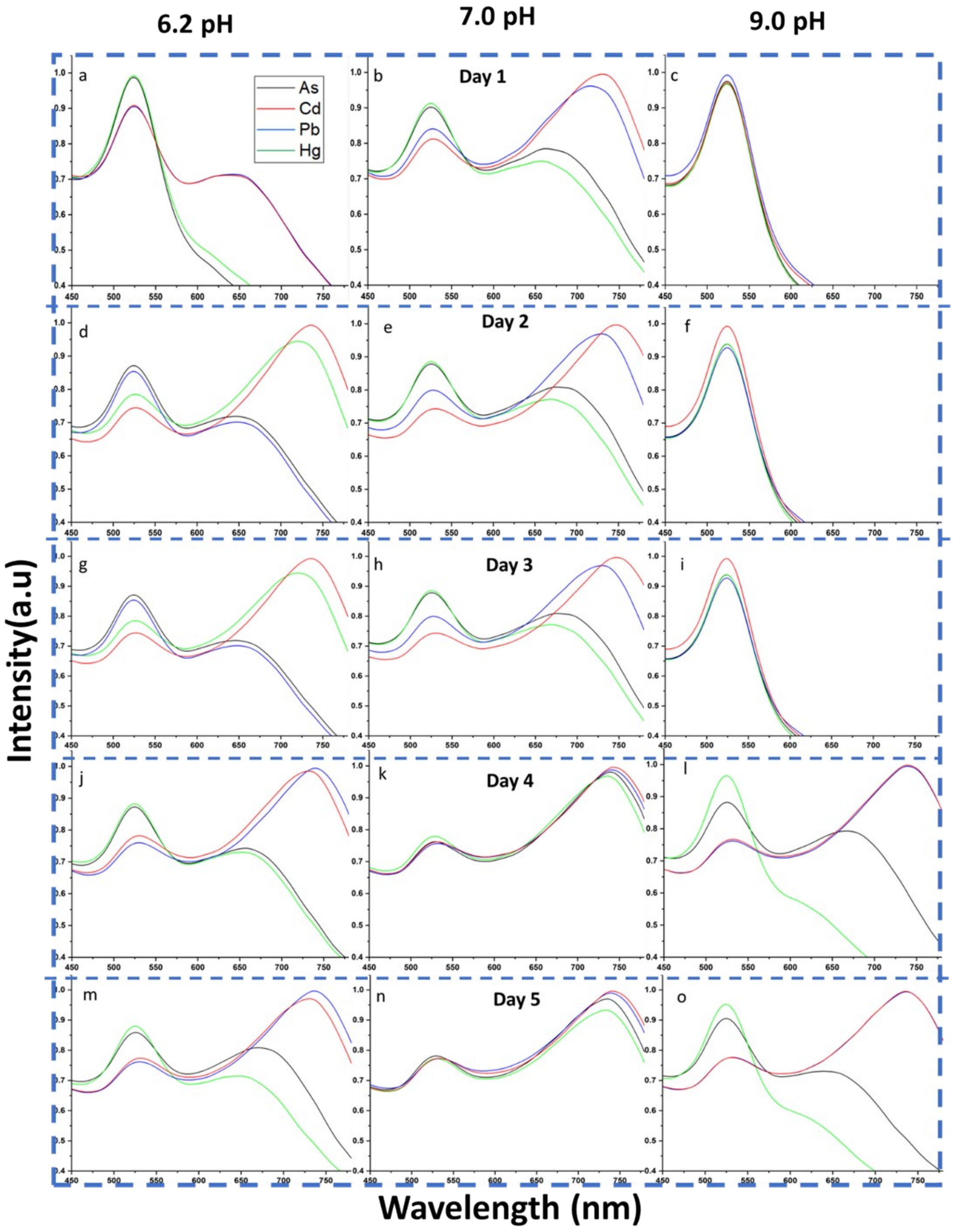

Supplementary Fig 3: Wavelength vs. intensity graph of gold nanoparticles of 31nm (AuNPs) functionalized with L-cysteine with three different pH's 6.2, 7.0 and 9.0 for 5 days. (First row, middle row and third row respectively) with heavy metals.

Supplementary Fig 4: Absorbance graph of AuNP (25 nm)-L Cys and heavy metals

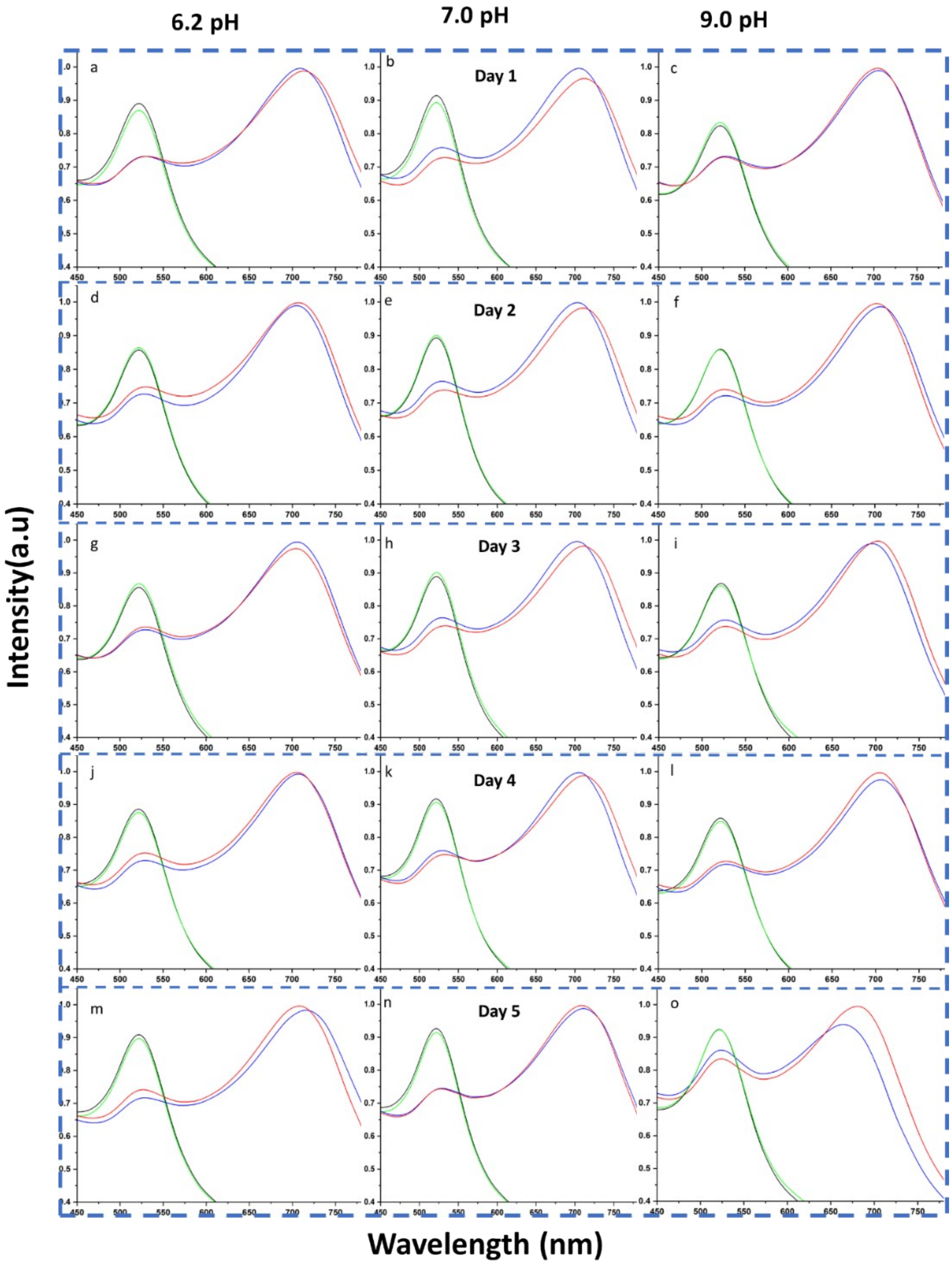

Supplementary Fig 4: Wavelength vs. intensity graph of gold nanoparticles (AuNPs) with 25nm functionalized with L-cysteine with three different pH's 6.2, 7.0 and 9.0 for 5 days. (First row, middle row and third row respectively) with heavy metal.
